# Inflammatory-induced spinal dorsal horn neurons hyperexcitability is mediated by P2X4 receptors

**DOI:** 10.1097/PR9.0000000000000660

**Published:** 2018-05-23

**Authors:** Franck Aby, Sara Whitestone, Marc Landry, Lauriane Ulmann, Pascal Fossat

**Affiliations:** a Institut Interdisciplinaire de Neurosciences (IINS), Université de Bordeaux, CNRS UMR5297, Bordeaux, France; b Institute of Functional Genomics, Université de Montpellier, Unité Mixte de Recherche 5302 CNRS, Montpellier, France, Unité de recherche U1191, INSERM, Montpellier, France, LabEx Ion Channel Science and Therapeutics.

**Keywords:** P2X4 Receptor, Inflammation, Dorsal horn neurons, Excitability

## Abstract

**Introduction:**

Purinergic ionotropic P2X receptors (P2RX) are involved in normal and pathological pain transmission. Among them, P2X4 are expressed in dorsal root ganglion and in the spinal cord. Their activation during nerve injury or chronic peripheral inflammation modifies pain sensitivity that leads to the phenomenon of allodynia and hyperalgesia.

**Objectives:**

We study here, in vivo, the role of P2X4 on the excitability of dorsal horn neurons (DHNs) in naive or pathological context.

**Methods:**

We recorded DHNs in vivo in anesthetized wild-type or P2RX4^−/−^ mice. We measured nociceptive integration and short-term sensitization by DHNs both in naive and inflamed mice.

**Results:**

Our results indicate that P2X4 alter neuronal excitability only in the pathological context of peripheral inflammation. Consequently, excitability of DHNs from inflamed P2RX4^−/−^ mice remains similar to naive animals.

**Conclusion:**

These results confirm the prominent role of P2X4 in inflammatory pain context and demonstrate that P2X4 are also involved in the hyperexcitability of DHNs.

## 1. Introduction

The dorsal horn of the spinal cord represents the first relay of pain transmission in the central nervous system. Peripheral afferent fibers contact different populations of dorsal horn neurons (DHNs) that in turn activate projection neurons.^11^ It has been demonstrated that changes of network activity in the dorsal horn of the spinal cord may explain the increase of pain behavior in pathological pain models.^9^ Purinergic signaling is involved in pain transmission, and P2X4 receptors (P2X4) are key players in the induction of chronic pain.^12^ Indeed, afferent nerve lesions induce activation of microglial P2X4 in superficial layers of the spinal cord leading to the modification of DHN excitability.^1,2^ Peripheral P2X4, expressed in macrophages and sensory neurons, are also involved in mechanical hypersensitivity in acute and chronic peripheral inflammation.^5–7,14,15^ However, the contribution of P2X4 to neuronal excitability in the spinal cord in naive or inflamed animals has not been yet investigated in vivo. In this study, we performed in vivo extracellular recordings in anesthetized mice to evaluate dorsal horn wide dynamic range (DH WDR) neuron excitability 24 hours after peripheral inflammation induced by Complete Freund Adjuvant (CFA) injection in wild-type (WT) and P2RX4^−/−^ mice.

## 2. Methods

### 2.1. Animals

Male mice, 6 to 12 weeks old, were maintained under a standard 12-hour light/dark cycle with food and water available ad libitum. All experiments followed European Union (Council directive 86/609EEC) and institutional guidelines for laboratory animal care. Institutional license for hosting animals was approved by the French Ministry of Agriculture (APAFIS#3751-2016030711446220 v2).

Mice were backcrossed for at least 20 generations and then maintained as separate P2X4 knockout (P2RX4^−/−^) and WT lines as described in Ref. 10. We defined 32 mice in this study separated in 4 different groups: 8 WT, 8 WT CFA, 8 P2RX4^−/−^, and 8 P2RX4^−/−^ CFA. According to Charan and Kantharia, this gives us an E value of 28, which is considered suitable for statistical analysis.^3^ We recorded a total of 76 DH WDR neurons (15 in WT, 15 in P2RX4^−/−^, 27 in WT CFA, and 19 in P2RX4^−/−^ CFA).

### 2.2. Induction of peripheral inflammation

Mice were subcutaneously injected with 10 μL of CFA (Sigma-Aldrich, Saint-Quentin Fallavier, France) in the dorsal part of the left hind paw. The efficiency of inflammation was assessed by measuring the edema 24 hours after injection and just before electrophysiological recordings. A picture of the paw was taken under graph paper, and the surface of the edema was measured with ImageJ software.

### 2.3. In vivo electrophysiology

**Figure 1.**
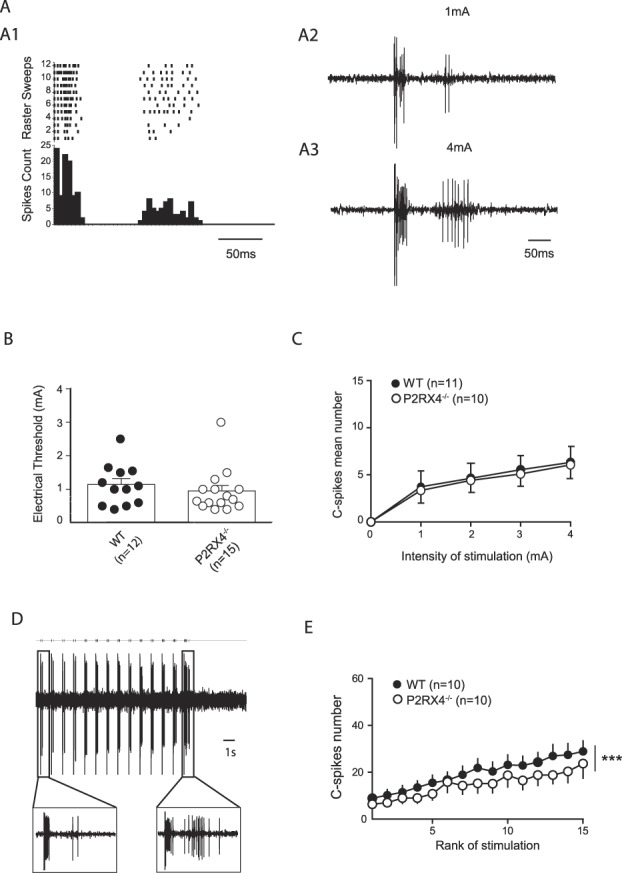
P2X4 do not change dorsal horn wide dynamic range (DH WDR) neurons excitability. (A) Representative dorsal horn neuron (DHN) response to nociceptive inputs. (A1) Poststimulus histogram and raster plot of DH WDR neurons. (A2) Response of DH WDR neurons to 1-mA stimulation of the paw. (A3) Response of DH WDR neurons to 4-mA stimulation of the paw. (B) Threshold for the C-fiber response of WDR neurons is similar in WT and P2RX4^−/−^ mice (1.12 ± 0.17 and 0.93 ± 0.17, n = 12 and 15, respectively, *P* = 0.28). (C) Intensity response curve is not changed in P2RX4^−/−^ mice as compared to WT (n = 11 and 10, respectively, *P* = 0.75). (D) Example of progressive increase in WDR discharge showing up a wind-up of the discharge. Note that number of spike C is higher after 14 stimulations as compared to the first stimulation. (E) Wind-up is lowered in P2RX4^−/−^ as compared to WT mice (n = 10 and 10, respectively, *P* < 0.001). WT, wild-type.

Mice were first anesthetized with isoflurane (3% induction and 1.5% maintenance) and placed on a stereotaxic frame (Unimécanique, Asnières, France). A laminectomy was performed on lumbar vertebrae T13 to L1, and segments L4 to L5 of the spinal cord were exposed. Extracellular recordings of DH WDR were made with borosilicate glass capillaries (2 MΩ, filled with NaCl 684 mM) (Harvard Apparatus, Cambridge, MA). The criterion for the selection of a neuron was the presence of a A-fiber-evoked response (0–80 ms) followed by a C-fiber-evoked response (80–300 ms) to electrical stimulation of the ipsilateral paw with bipolar electrodes connected to a stimulator and placed in the center of the DH WDR (see Poststimulus Histogram, Fig. 1). The threshold for C-fiber-evoked response was first evaluated. Then, the intensity response (IR) curve was performed by progressively increasing the intensity of electrical stimulation of the sciatic nerve (1–4 mA). Wind-up was elicited with a train of 15 stimuli (1-Hz frequency), 3-fold over the threshold for the C fiber. We recorded at least one neuron per animal.

### 2.4. Statistical analysis

Results are expressed as mean ± SEM. The C-fiber threshold for each experimental group was compared using nonparametric Mann–Whitney test. Intensity response curves were analyzed using 2-way analysis of variance (ANOVA) followed by a Bonferroni post-test. Wind-up curves were analyzed using 2-way ANOVA. Comparison of edema was made using a 1-way ANOVA followed by a Bonferroni post-test. A *P* value < 0.05 was considered as significant.

## 3. Results

Peripheral electrical stimulation of the center of the receptive field of DH WDR elicits the response from fast (within the first 50 ms) nonnociceptive A fibers and slow (around 150 ms) nociceptive C fibers (Fig. 1A1–A3). We measured the threshold for the appearance of the C-fiber response, the IR curve, ie, the mean number of C spikes induced by peripheral electrical stimulations in both WT and P2RX4^−/−^ mice (Fig. 1B–C). We observed no difference in the C-fiber threshold between WT and P2RX4^−/−^ mice (Fig. 1B). The IR was not altered by the knockout of P2X4 (Fig. 1C). Dorsal horn WDR can generate a form of short-term sensitization called wind-up: progressive neuronal discharge in response to repetitive stimulations of the same intensity and at low frequency (Fig. 1D). We evaluated the proportion of DH WDR expressing wind-up, and we found no difference between WT and P2RX4^−/−^ mice (10/15 and 10/17, respectively). The amplitude of wind-up was significantly different in WT and P2RX4^−/−^ mice (Fig. 1E).

**Figure 2.**
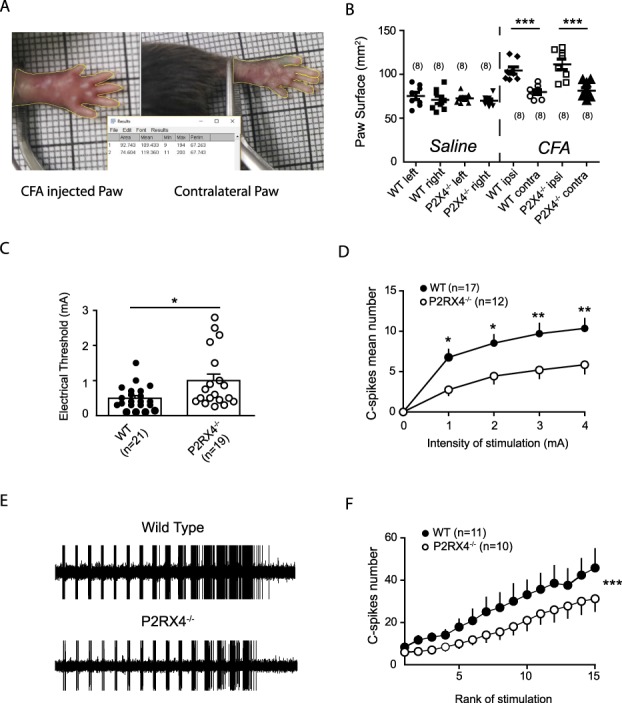
P2X4 is involved in Complete Freund Adjuvant (CFA)–induced hyperexcitability of WDR neurons. (A) Injection of CFA induces an edema in the injected paw 24 hours after injection. (B) Surface of the injected paw is significantly bigger than the contralateral noninjected paw (79.77 ± 2.75 mm^2^ contralateral vs 104 ± 4 mm^2^ ipsilateral, n = 8, *P* < 0.001 for WT CFA; 81.3 ± 3.4 mm^2^ ipsilateral 111.3 ± 5.9 mm^2^ contralateral for P2RX4^−/−^ CFA, n = 8, *P* < 0.001). No difference is observed in the level of the edema between WT and P2RX4^−/−^ mice. (C) Threshold for the C-fiber response of WDR neurons is significantly decreased in WT mice compared with P2RX4^−/−^ (0.49 ± 0.07 [WT] and 1 ± 0.2 [P2RX4^−/−^], n = 21 and 19, respectively, *P* = 0.02). (D) Intensity response curve is significantly lower in P2RX4^−/−^ mice (*P* < 0.001). From 1 to 4 mA, mean number of C spike are significantly smaller in P2RX4^−/−^ than in WT mice (Bonferroni post-test, *P < 0.05, **P < 0.01). (E) Amplitude of wind-up is decreased in P2RX4^−/−^ mice as compared to WT mice (n = 10 and 11, respectively, *P* < 0.001). WT, wild-type.

To determine whether P2X4 are involved in DH WDR excitability in the context of pathological pain, we performed the same analyses on WT and P2RX4^−/−^ mice 24 hours after the plantar injection of CFA. We first evaluated the level of inflammation by measuring the surface of the injected paw (Fig. 2A–B). Twenty-four hours after the injection of CFA, the plantar surface of the injected paw significantly increased in both WT and P2RX4^−/−^ mice (Fig. 2B). Next, we evaluated the excitability of DHNs after inflammation in both wild-type and P2RX4^−/−^ mice and compared the results with the naive conditions. We observed that CFA significantly decreased the threshold for C fibers (*P* = 0.0012, not shown). Dorsal horn WDR from P2RX4^−/−^ mice presents a significantly higher threshold for C fibers when compared with WT mice after CFA injection (Fig. 2C) that remained similar to naive P2RX4^−/−^ (*P* = 0.61, not shown). Similarly, the IR curve is also significantly different between WT CFA and P2RX4^−/−^ CFA mice (Fig. 2D). We then measured the amplitude of the wind-up in inflammatory condition. Although the same proportion of DH WDR expresses wind-up in both WT CFA and P2RX4^−/−^ CFA mice (11/20 and 10/16, respectively), inflammation in WT mice induces a significant increase of the wind-up when compared with naive condition (*P* < 0.001, not shown). Interestingly, this increase in wind-up amplitude in inflammatory conditions is absent in P2RX4^−/−^ CFA mice when compared with WT CFA (Fig. 2E).

## 4. Discussion

In this study, we report that P2X4 is not involved in acute nociceptive transmission but participate in central sensitization of DH WDR in both naive and the persistent pain model.

Complete Freund Adjuvant is known to produce a fast onset and long-lasting inflammation that elicits allodynia and hyperalgesia, both symptoms of the chronic pain state. Previous studies have demonstrated that behavioral changes seem very early after CFA injection.^13,14^ We show here that these behavioral changes are also accompanied by an alteration of nociceptive integration by DH WDR neurons. Indeed, 24 hours after CFA injection, hyperexcitability of DH WDR neurons is observed through a decrease in the C-fiber response threshold and an increase of the DH WDR neuron responsiveness to electrical stimulation within their receptive field. These alterations are perhaps due to peripheral changes that could affect nociceptive fibers by increasing their activation threshold and therefore their responsiveness to electrical stimulation. Indeed, a previous study already demonstrated an increased excitability in peripheral fibers in chronic inflammatory pain.^16^ Central mechanisms can also be responsible for such changes in DH WDR neurons. Superficial laminas of the spinal cord receive most C fibers and are connected to DH WDR neurons.^11^ It is well known that increased excitability of superficial lamina also occurs in the model of persistent pain.^2^ Wind-up is a form of sensitization to pain that is not expressed by peripheral fibers and thus restricted to the dorsal horn. Complete Freund Adjuvant induced an increase in wind-up amplitude suggesting that both central and peripheral mechanisms are responsible for the increased excitability of DH WDR. In addition, wind-up expressed by DH WDR can also be elicited by intrinsic properties that depend on L-type calcium channels.^4,8^ Therefore, we cannot rule out a direct effect of inflammation on the intrinsic properties of DH WDR.

Our results show that DH WDR hyperexcitability induced by inflammation is largely suppressed in P2RX4^−/−^ mice. These results are in line with previous studies showing a P2X4-dependant mechanical hyperalgesia 24 hours after CFA injection.^5,14^ We show that P2RX4 knockout alters the C-fiber threshold and the IR curve that suggests a peripheral role of P2X4. This is in accordance with the involvement of peripheral P2X4 in persistent pain models.^5–7,14,15^ Finally, wind-up amplitude of DH WDR is decreased in P2RX4^−/−^ CFA mice, which also suggests a central component.

Finally, we demonstrated that CFA-induced chronic inflammation altered nociceptive integration by DH WDR neurons as early as 24 hours after CFA injection, and that both peripheral and central mechanisms are mediated by P2X4.

## Disclosures

The authors state that they have no conflict of interest.

This work was funded by the Languedoc-Roussillon “Chercheur d'avenir program,” and Institut UPSA de la douleur. This work was also funded by the LABEX Brain.
